# 

**Published:** 1880-12

**Authors:** 


### Table of Contents.

ORIGINAL COMMUNICATIONS.

An Unusnal Wound Complication Incident to an Operation for Congenital Phimosis. By Dr. S. N. Jordan, Columbus, Ga....... 513

REPORTS OF SOCIETIES.

Michigan State Board of Health........ 514
Proceedings of the Suffolk District Medical Society........ 518
New York Academy of Medicine........ 526
New York Society of German Physicians........ 531
Minutes of the Biological and Microscopical Section of the Academy of Natural Sciences, Nov. 1st, 1880........ 536

SELECTIONS.

A Case of Dropsy. By J. F. Goldman, M.D., Huntsville, Ala........ 541
Chian Turpentine, and its use in Cancer........ 542
Clinical Lecture. Pleurisy, Pneumonia, Disease of the Heart. By Austin Flint, M D........ 550
Antagonism of Remedies........ 564

EDITORIAL.

Close of the Year........ 568
Meteorological Report for November........ 569

BIBLIOGRAPHICAL.

The Cure and Culture of Children........ 570

Practical Treatise on Surgical Diagnosis........ 570

Diagnosis and Treatment of Ear Diseases........ 570

A Treatise on Diphtheria........ 571

Pamphlets received........ 572

EXCERPTA.

Humorous Physiology........ 573
Indican in the Urine of Typhoid Patients........ 575
Mark Twain's Receipt for New England Pie........ 576
Test for Arsenic........ 576
Hay Fever........ 576
Vaccine Virus........ 576

